# Analysis of Leukocyte Recruitment in Continuous Veno-Venous Hemofiltration with Regional Citrate vs. Systemic Heparin Anticoagulation

**DOI:** 10.3390/cells11111815

**Published:** 2022-06-01

**Authors:** Andreas Margraf, Chang Liu, Mira Küllmar, Melanie Meersch, Jan Rossaint, Alexander Zarbock

**Affiliations:** 1 Department of Anesthesiology, Intensive Care and Pain Therapy, University Hospital Münster, 48149 Münster, Germany; margrafa@uni-muenster.de (A.M.); liuchangccm@hospital.cqmu.edu.cn (C.L.); mira.kuellmar@ukmuenster.de (M.K.); meersch@uni-muenster.de (M.M.); rossaint@uni-muenster.de (J.R.); 2 The Department of Critical Care Medicine, The First Affiliated Hospital of Chongqing Medical University, 400016 Chongqing, China

**Keywords:** acute kidney injury, CVVH, anticoagulation, citrate, heparin, leukocytes, inflammation

## Abstract

Acute kidney injury (AKI) is a frequent complication in critically ill patients. Supportive treatment of AKI patients is based on renal-replacement therapy, including continuous veno-venous hemofiltration (CVVH). To limit clotting events on extracorporeal surfaces, anticoagulants are administered, including systemic heparin and local citrate. The differential and comparative effects of these anticoagulants on leukocyte function in acute kidney injury patients are, so far, insufficiently understood. In this bio-add-on-study, AKI patients were randomized as part of a parallel-group trial to either systemic heparin or regional citrate anticoagulation. Patient samples were collected upon inclusion, prior to CVVH initiation at day 0, day 1, day 3 and day 5, following CVVH initiation, and one day after cessation of CVVH, then immediately analyzed. Flow cytometric assessment of surface-receptor molecules was conducted. Whole-blood-perfused human microfluidic chambers were used for the analysis of neutrophil rolling and adhesion. Acute kidney injury was associated with significant changes in the surface expression of CD182 and CD16 throughout CVVH treatment, independent of the anticoagulation regime. AKI furthermore abrogated selectin-induced slow leukocyte rolling and diminished chemokine-induced leukocyte arrest. Subgroup analyses of citrate vs. heparin treatment showed no significant differences between groups, independent of the duration of CVVH treatment. CD182 and CD16 expression remained low in both groups throughout CVVH therapy. These data confirm that AKI impairs selectin-mediated leukocyte slow rolling and chemokine-induced leukocyte arrest in vitro. Systemic heparin or local citrate anticoagulation have no differential effect on the leukocyte recruitment steps examined in this study.

## 1. Introduction

Acute kidney injury (AKI) is associated with high mortality rates in hospitalized patients [1]. Therapeutic options include renal-replacement therapy (RRT), which, according to a recent network meta-analysis, reduces mortality in septic AKI patients [2]. Continuous veno-venous hemofiltration (CVVH) is commonly used in critically ill patients, whereas the timing of initiation of RRT is discrepantly reported in the literature, but appears to have no effect in severe AKI [3,4]. To prevent filter clotting and prolong CVVH treatment, appropriate anticoagulation is required. The two main procedures for anticoagulation in CVVH are systemic heparin or regional citrate application. Risks of anticoagulants include bleeding and adverse effects, such as heparin-induced thrombocytopenia or citrate-induced electrolyte- and pH-derangements. Former studies point towards a benefit of regional citrate anticoagulation compared to the systemic administration of heparin [5,6], and The Kidney Disease: Improving Global Outcomes (KDIGO) guidelines recommend usage of regional citrate anticoagulation for continuous renal-replacement therapy [7]. A meta-analysis concluded that mortality is not affected by heparin versus citrate treatment, but citrate appears to be more successful in the prolongation of circuit life span and reducing bleeding events [8]. A recent multicenter study equally concluded that regional citrate is favorable in the prolongation of filter life span compared to systemic heparin [9]. Nonetheless, substituting the filtration capacity of a patient’s kidney by utilization of artificial-surface-based RRT results in activation of the complement system and different cell types, including platelets and leukocytes [6,10].

Leukocytes, most notably neutrophils, are known for their function in immune defense against invading pathogens. The recruitment of these cells follows a simplified cascade-like pattern. In this, leukocytes roll along activated endothelial cells, a process reliant on endothelial cell-expressed E- and P-selectin and leukocyte-expressed CD162 (PSGL-1). The interaction of integrins, namely CD11a/CD18 (Leukocyte function antigen-1, LFA-1) and CD11b/CD18 (Macrophage-1 antigen, Mac-1) with the intercellular adhesion molecule (ICAM)-1 further reduces the leukocyte rolling velocity. Chemokine stimulation (e.g., by interleukin-8 binding to CD182 on leukocytes) induces the high-affinity conformation of the integrin and the subsequent arrest of the cell [11]. Alterations in these recruitment steps result in an inflammatory imbalance, evoking either an increased risk of infections or self-harm through autoimmunity [12]. Interestingly, we recently observed a significantly higher rate of infections in critically ill patients experiencing AKI randomized to regional citrate anticoagulation treatment in comparison to systemic heparin administration, but reasons for this are, so far, incompletely understood [9].

Thus, the aim of this study was to examine the effects of AKI and different modalities of anticoagulation during CVVH on neutrophil recruitment.

## 2. Materials and Methods

### 2.1. Reagents

All reagents were obtained from Sigma Aldrich (Taufkirchen, Germany), unless otherwise stated.

### 2.2. Study Protocol

The study was a single-center bio-add on study of a previously published study protocol [13]. Briefly, the main study was a randomized multicenter, parallel-group clinical trial, approved by the Federal Institute for Drugs and Medical Devices (BfArM, EudraCT-No. 2014-004854-33) and registered at clinicaltrials.gov (NCT02669589). Consent for participation from all patients prior to randomization was obtained. Inclusion criteria for patients were: KDIGO stage 3 acute kidney injury classification, at least one additional condition (severe sepsis or septic shock, use of vasopressor, refractory fluid overload), age between 18 and 90 years, intention to provide full intensive care therapy for at least 3 days and written informed consent. Exclusion criteria included increased risk of bleeding, diseases with hemorrhagic diathesis, requirement of therapeutic anticoagulation, previous allergic reactions to one of the anticoagulants, known history of heparin-induced thrombocytopenia, severe lactic acidosis in the context of acute liver failure and/or shock, dialysis-dependent chronic kidney disease, permanent occlusion or surgical lesion of both kidney arteries as cause for AKI, (glomerulo-)nephritis as AKI cause, interstitial nephritis, vasculitis, urinary tract obstruction, kidney transplant within the last 12 months, hemolytic–uremic syndrome/thrombotic thrombocytopenic purpura, unavailability of machine for CVVH at inclusion, participation in another clinical intervention trial in last 3 months, any persons with dependency on the investigator, pregnancy or impending miscarriage. Randomization for the main study was performed by the Clinical Trials Centre Leipzig in a 1:1 proportion using a minimization method with random component for balanced assignment, as described in the RICH-trial [9].

Following inclusion, RRT was started within 24 h after meeting KDIGO stage 3 criteria. In the systemic-anticoagulation-with-heparin group, target was an activated partial thromboplastin time of 45–60 s. In the regional citrate anticoagulation group, continuous citrate addition to the blood before the filter of the extracorporeal circuit was performed in dependence on ionized calcium levels. For further details, please see Zarbock et al. [9]. Samples for laboratory measurements were collected as indicated, transferred to the laboratory and immediately processed. Sample collection, patient inclusion in the bio-add-on study and measurements at different time points were dependent on availability of laboratory capacities.

### 2.3. Blood-Perfused Human Micro-Flow Chamber

To analyze selectin-mediated neutrophil rolling of patient samples, we used a previously described whole-blood-perfused human microfluidic chamber system [14]. In this model, it has been shown that above 90% of all rolling cells are polymorphonuclear neutrophils (PMNs) [15]. Briefly, the chambers were coated with E-selectin (2 μg/mL) (R&D Systems, Minneapolis, MN, USA), P-selectin (15 μg/mL) (R&D Systems, Minneapolis, MN, USA), E-selectin/ICAM-1 (2/6 μg/mL) (R&D Systems, Minneapolis, MN, USA) or P-selectin/ICAM-1 (15/6 μg/mL) (R&D Systems, Minneapolis, MN, USA) for 2 h. The chambers were then blocked with 1% casein (Fisher Scientific, Waltham, MA, USA) for 1 h and subsequently perfused with heparinized whole blood at a constant shear stress of 5–6 dynes/cm^2^. For analysis of chemokine-induced arrest of rolling PMNs, flow chambers were coated with P-selectin (15 μg/mL), ICAM-1 (6 μg/mL) and Interleukin-8 (IL-8) (50 μg/mL, Peprotech, Rocky Hill, NJ, USA) or P-selectin and ICAM-1 as control. After two minutes of perfusion with heparinized whole blood at a shear stress of 5–6 dynes/cm^2^, rolling and adhering cells were counted per field of view. 

### 2.4. Flow Cytometric Analysis of Neutrophils from Human Whole Blood

Flow cytometry was used to analyze surface expression of adhesion molecules on human neutrophils. Cells were isolated using a Histopaque^®^ density gradient (Sigma Aldrich, Taufkirchen, Germany), washed and incubated with according anti-human antibodies. Antibodies included: anti CD11b, clone M1/70; anti CD14, clone HCD14; anti CD15, clone HI98; anti CD49d, clone 9F10 (all from BioLegend, San Diego, CA, USA); anti CD182, clone 6C6; anti CD 29, clone HUTS-21; anti CD162, clone KPL-1 (all from BD Bioscience, Franklin Lakes, NJ, USA); anti CD11a, clone MEM-25; anti CD62L, clone LT-TD180 (all from ImmunoTools, Friesoythe, Germany). Samples were measured on a BD FACS Canto 2 flow cytometer and data were analyzed using FlowJo v7.1.

### 2.5. Statistical Analysis

Data were entered into Microsoft Excel and subsequently analyzed using GraphPad Prism v.6 and Systat SigmaPlot v12. Data were assessed for normality using Shapiro–Wilk test, and power analyses were performed in SigmaPlot v12.0. Two groups were analyzed by two-tailed t-test or Mann–Whitney Rank Sum test, respectively. Multi-group analyses were performed using ANOVA with post hoc testing (Tukey’s method). Rolling-flow chamber experiments in AKI vs. control group were analyzed by *t*-test. The main goal of this analysis was to show a reduction in rolling velocities between selectin only and selectin + ICAM-1 in each condition. In subsequent analyses, we then focused only on differences in selectin vs. selectin and selectin + ICAM-1 vs. selectin + ICAM-1 conditions. Further multi-group rolling-flow chamber experiments were thus analyzed per condition using ANOVA (E-selectin control vs. E-selectin heparin vs. E-selectin citrate; E-selectin + ICAM-1 control vs. E-selectin + ICAM-1 heparin vs. E-selectin + ICAM-1 citrate). Adhesion flow chambers were analyzed with ANOVA. A *p* ≤ 0.05 was considered statistically significant. 

## 3. Results

### 3.1. Patient Characteristics

Patients were screened for appropriate inclusion criteria, including AKI, and included as described above. Patient characteristics are described in Table 1. Control samples were obtained from healthy volunteers.

### 3.2. Effects of AKI and Mode of Anticoagulation on Neutrophil Surface Expression Characteristics

**Figure 1:**
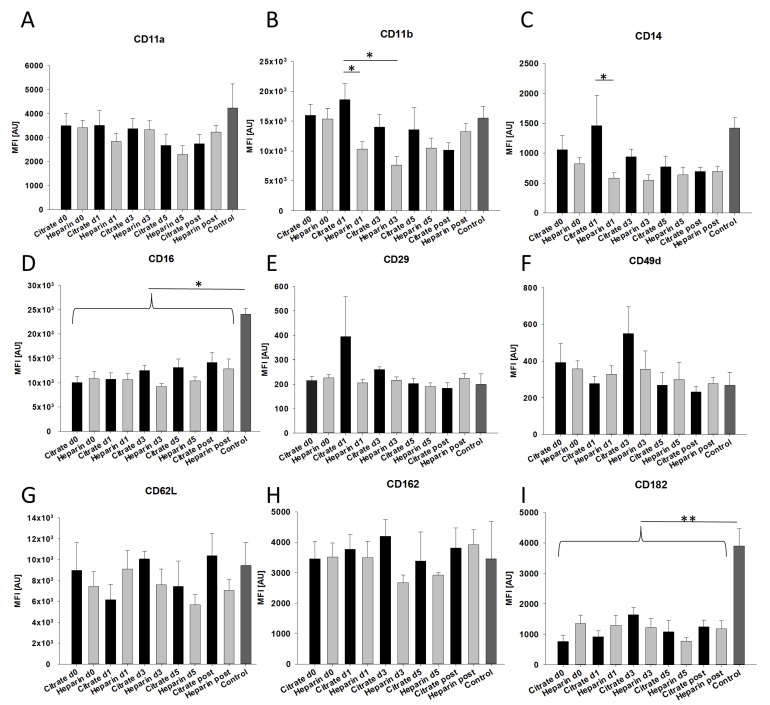
Neutrophil surface expression. Levels of important neutrophil surface molecules (CD11a (**A**), CD11b (**B**), CD14 (**C**), CD16 (**D**), CD29 (**E**), CD49d (**F**), CD62L (**G**), CD162 (**H**), CD182 (**I**)) were measured by flow cytometry in density-gradient-isolated neutrophils from critically ill patients experiencing acute kidney injury before (d0), during (d1, d3, d5) and after (d1 post) either regional-citrate- or systemic-heparin-based continuous veno-venous hemofiltration and from healthy volunteers (control). Mean fluorescence intensity (MFI) measurements of CD11b and CD14 differed between citrate and heparin groups. CD16 and CD182 surface levels were all significantly reduced in comparison to healthy controls. (n ≥ 3 individual patients; * *p* < 0.05; ** *p* < 0.01; mean ± SEM).

Leukocyte function and activation depend on the expression of relevant surface molecules involved in inflammation. These include, amongst others, CD11a (integrin LFA-1), CD11b (integrin Mac-1), CD14, which was shown to impact LPS-dependent systemic inflammation [16], CD16, which was linked to neutrophil apoptosis [17], CD162 (PSGL-1) and CD182 (CXCR2, chemokine receptor). Loss of any of these receptors is known to result in impaired leukocyte recruitment and/or functionality [18,19]. To examine effects of acute kidney injury and different modes of anticoagulation on the expression of relevant surface molecules, we performed flow-cytometry-based measurements of density-gradient-isolated neutrophils. When comparing overall expression levels, significant differences were found in CD11b (citrate day 1 vs. heparin day 1 and citrate day 1 vs. heparin day 3), CD14 (citrate day 1 vs. heparin day 1), CD16 (all vs. control) and CD182 (all vs. control) (Figure 1).

**Figure 2:**
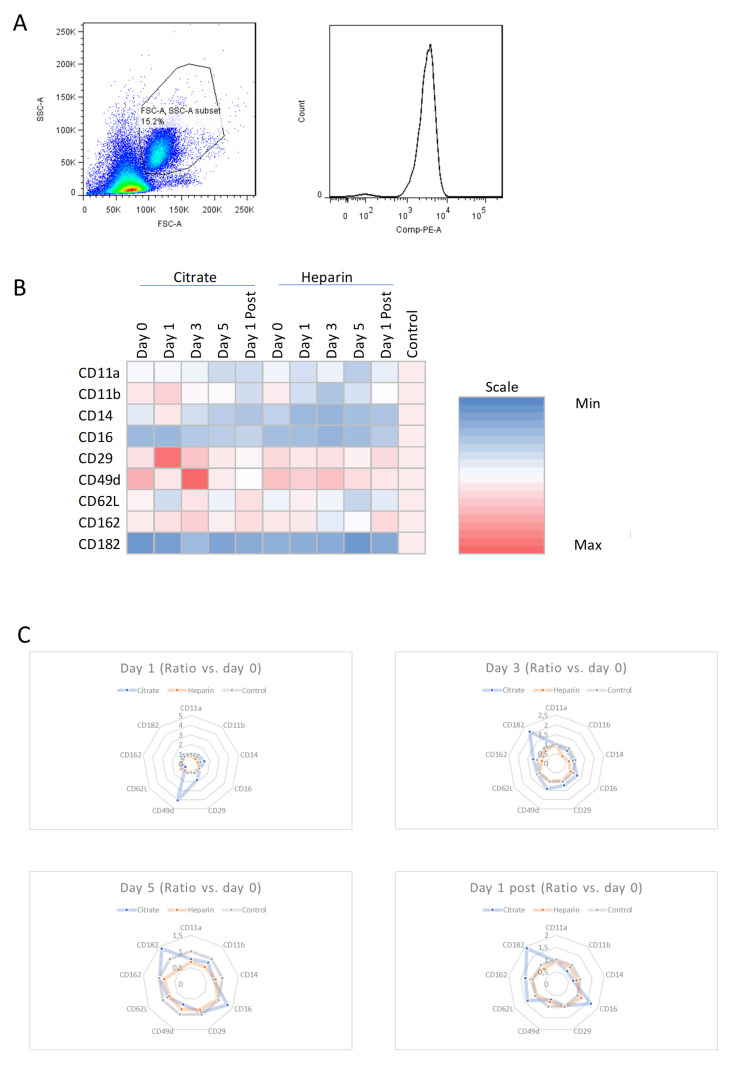
Leukocyte phenotype profiles. (**A**) Representative gating strategy of flow cytometry data is shown for determination of neutrophils. (**B**) Heat-map representation of expression levels indicative of leukocyte phenotype profiles relative to healthy control expression levels. (**C**) Spider-graph representation of expression levels relative to group-expression levels before CVVH initiation (d0).

An exemplary FACS-gating strategy is presented in Figure 2A. Leukocyte phenotype changes can be observed based on not only one, but a combination of multiple surface characteristics [20]. When focusing on ratiometric phenotype profiles of leukocytes, the heatmap representation obtained by calculating the ratio of the according mean fluorescence intensity of each sample, divided by the overall control mean fluorescence intensity, shows distinct leukocyte expression patterns in AKI patients and throughout CVVH treatment. Spider-graph representations are displayed to exemplify multiparametric phenotype changes due to different CVVH treatments, thus showing the expression patterns in relation to surface expression of the according group on day 0 (prior to initiation of CVVH), further hinting towards only minor regulatory effects during CVVH in AKI patients (Figure 2B,C).

### 3.3. Selectin-Mediated Slow Leukocyte Rolling Is Abolished in Patients with AKI

**Figure 3:**
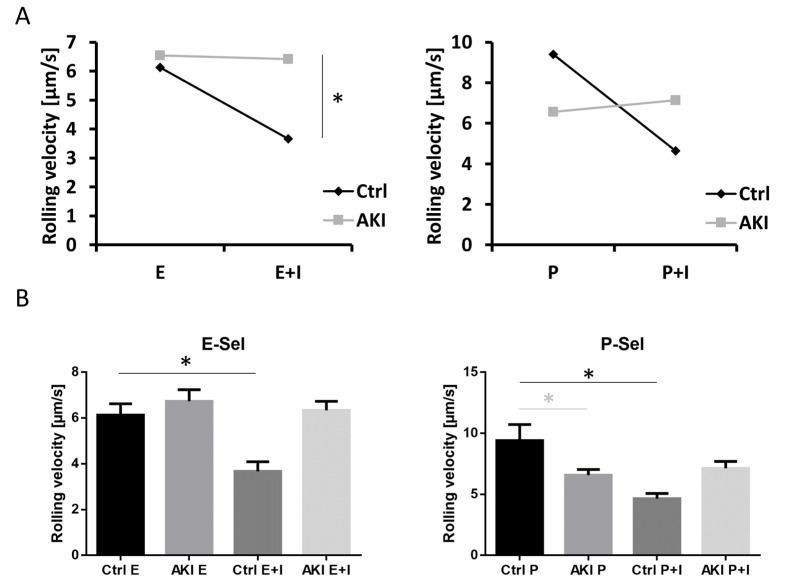
Acute kidney injury impairs leukocyte slow rolling. (**A**) Slow leukocyte rolling was assessed using whole blood perfused into microfluidic chamber devices. (**B**) Bar-graph representation with error bars of (**A**): *T*-test was performed for analysis of integrin response for each condition (shown as black bar and asterisk). Inter-group variations were tested using ANOVA (light gray bar and asterisk) (n ≥ 3 individual patients; * *p* < 0.05; mean ± SEM).

As relevant interaction (CD162, PSGL-1) and adhesion (CD11a, CD11b) molecule expression was not different between AKI (day 0 groups, prior to CVVH initiation) and control samples, we next focused on functional impairments of AKI patient-derived leukocytes. For this purpose, we used the model of whole-blood-perfused in vitro flow chambers, in which blood samples were perfused through small glass capillaries coated with indicated proteins resembling the surrounding of activated endothelial cells. Interaction of leukocyte-expressed CD162 with E- or P-selectin leads to leukocyte rolling. Further interaction with the CD11a/b ligand ICAM-1 leads to a reduction in the leukocyte rolling velocity, as can be observed in the control samples. As reported previously [21], we observed an impaired leukocyte slow rolling response in AKI patients (Figure 3). Interestingly, the rolling velocity on P-selectin was significantly lower in the AKI group.

### 3.4. Mode of Anticoagulation and Duration of CVVH Have No Impact on Slow Leukocyte Rolling

**Figure 4:**
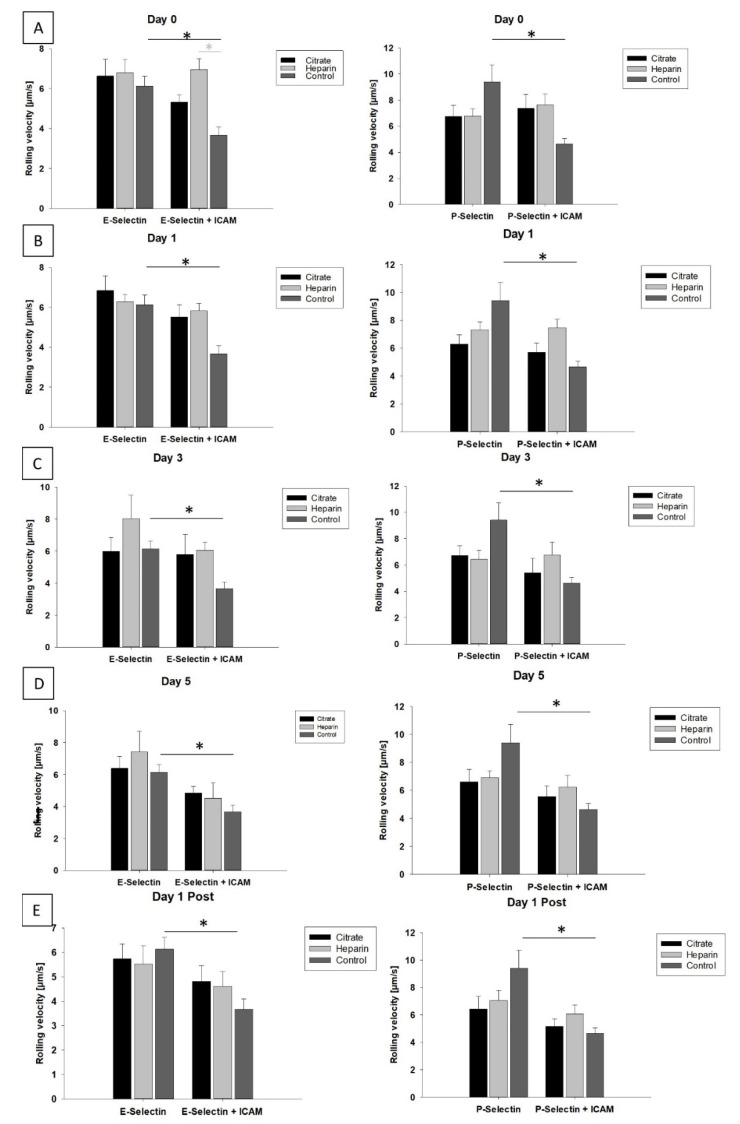
Leukocyte slow rolling in regional-citrate- and systemic-heparin-based CVVH. Sub-group analyses for systemic heparin vs. regional citrate slow leukocyte rolling were performed on day 0 (**A**), day 1 (**B**), day 3 (**C**), day 5 (**D**) and day 1 after CVVH discontinuation (**E**) and revealed no significant difference. *T*-test was performed for analysis of integrin response for each condition (shown as black bar and asterisk), where only the control subjects exhibited a significant ICAM-1-dependent reduction in rolling velocity, whereas this slow rolling response was not present in AKI patients. Light gray significance level in graphs represents results of in-group (either heparin vs. citrate vs. control in E-selectin or heparin vs. citrate vs. control in E + ICAM-1) analyses, tested by ANOVA. (n ≥ 3 individual patients; * *p* < 0.05; mean ± SEM).

To assess whether differences in infection rates might be due to CVVH-based anticoagulation regimens affecting leukocyte recruitment, we focused on the analysis of microfluidic chamber experiments of patients randomized to either regional citrate or systemic heparin protocols for CVVH and a healthy control population. For this purpose, we used data obtained by utilization of microfluidic chambers coated with E- or P-selectin with and without ICAM-1, again resembling the transition from rolling to slow rolling during the leukocyte recruitment cascade. Herein, we noted a significant difference on day 0 between control and heparin groups in the E-selectin + ICAM-1 condition, but no further significant variations according to mode of anticoagulation or duration of CVVH amongst groups. Only the control group showed the aforementioned significant reduction in rolling velocity between the E-selectin and E-selectin + ICAM-1 and P-selectin/P + ICAM-1 condition, respectively, exposing that CVVH does not resolve the leukocyte recruitment defect, independent of the anticoagulation strategy or duration of CVVH (Figure 4).

### 3.5. Chemokine-Induced Arrest Is Diminished in AKI Patients Independent of Mode of Anticoagulation or Duration of CVVH

**Figure 5:**
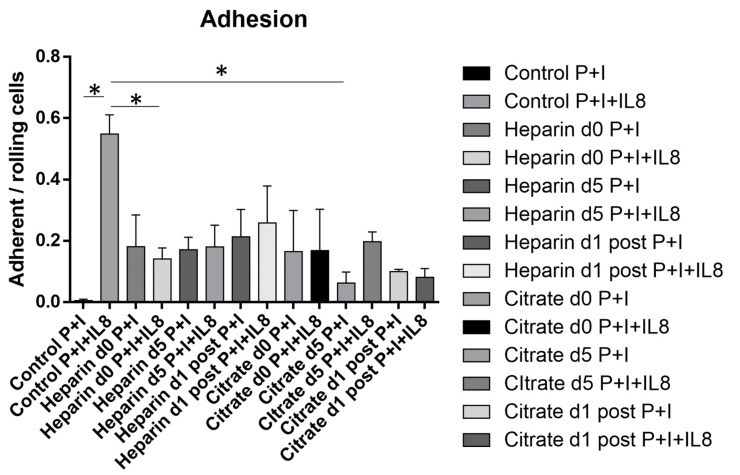
Chemokine-induced leukocyte adhesion. For analysis of chemokine-induced adhesion, whole blood was perfused into microfluidic chambers coated with either P-selectin and ICAM-1 or P-selectin, ICAM-1 and interleukin-8, and the ratio of adhering to rolling leukocytes determined. Control subjects exhibited a significant increase in ratio of adherent to rolling cells, whereas this increase was absent in all AKI-groups, independent of mode or duration of CVVH. (n ≥ 2 individual patients; * *p* < 0.05; mean ± SEM).

Following slow leukocyte rolling, the leukocyte recruitment cascade progresses to chemokine-mediated cell adhesion. As we found that AKI affected the expression of CD182, we next assessed functional consequences by stimulating whole-blood-perfused leukocytes with the chemokine interleukin-8 and measuring leukocyte adhesion. We again used the microfluidic chamber system and coated the chambers with either P-selectin and ICAM-1 alone, or with P-selectin, ICAM-1 and interleukin-8. Under control conditions, only a few cells adhered in P + I chambers, whereas the ratio of adherent to rolling cells significantly increased in P + I + IL8-coated control chambers. Focusing on the chemokine-induced arrest response in AKI/CVVH groups, we noted that the significant increase in adherent cells upon chemokine stimulation, as could be observed in the control group, was absent among all treatment groups. This observation was independent of mode of anticoagulation or duration of RRT, corroborating the functional relevance of reduced CD182 levels (Figure 5).

## 4. Discussion

Progressing acute kidney injury in critically ill patients demands substitution of the kidneys’ excretory function by renal-replacement therapy. To prevent clotting and filter dysfunction on these extracorporeal artificial surfaces, anticoagulation is needed. The commonly used anticoagulants for this procedure include the systemic administration of heparin or the regional addition of citrate, as the KDIGO guidelines recommend favored usage of regional citrate [7]. Recently, a large randomized parallel-group trial revealed increased susceptibility to infections in patients receiving regional-citrate-based CVVH protocols in comparison to systemic administration of heparin [9]. Reasons for this finding are, so far, unclear. Using microfluidic and flow cytometric laboratory techniques, we here confirm that acute kidney injury impairs the recruitment of leukocytes. Nonetheless, this effect is not impacted by CVVH treatment or choice of anticoagulant during renal-replacement therapy. 

Ex vivo assessment of leukocyte rolling and adhesion is a potent assay for measuring leukocyte in vivo functionality [14]. Indeed, previous work showed that AKI is associated with impaired leukocyte recruitment due to dysfunctional selectin-mediated signaling, and reproduced similar results in mice in vivo [21]. Our study confirms these data, showing that selectin-mediated neutrophil slow rolling is abrogated during AKI. Of note, the former study included animal data which showed no effect of AKI on chemokine-induced arrest, but no expression analysis of the functionally relevant receptor CD182 (CXCR2) was performed [21]. Our data now extend these previous reports, indicating that CD182 levels are significantly reduced in critically ill patients experiencing AKI, and could not be restored by renal-replacement therapy. Importantly, this defect directly translates to a functional impairment in leukocyte adhesion, following stimulation with interleukin-8, as could be visualized in microfluidic chamber devices. Reasons for this finding are, so far, unclear. It is known that CD182 surface levels are controlled by internalization upon ligand binding and subsequent recycling, but also by cleavage via a disintegrin and metalloprotease-17 (ADAM17). The induction of ADAM17 irreversibly downregulates CD182 receptor levels, thus mediating chemokine-based cellular functions [22]. Indeed, it was found that in murine polymicrobial sepsis, ADAM17 controls neutrophil recruitment and that ADAM17 levels and/or activity are elevated in sepsis models and in renal disease [23,24,25]. Interestingly, as ADAM17 also cleaves CD62L and CD16b, we noted no difference in CD62L levels, but equally reduced CD16 levels, which again hints towards increased ADAM17 activity or ADAM17-levels. Nonetheless, reduced CD16 levels have also been implicated as a marker for neutrophil apoptosis [17]. One limiting factor of note is that no ADAM17 activity was measured in our patient cohort, thus causality of the observed changes in surface expression characteristics remains yet to be determined. As another limitation, age and sex differences must be taken into consideration when interpreting the present data, as age and sex differed significantly between control and AKI subjects, and functional regulations, termed “immunosenescence” or “inflammaging”, are known with regard to the aging leukocyte, including reduced expression of CD182 in aged mice. Indeed, such age-dependent differences in CD182 expression are even more pronounced in a traumatic setting [26,27]. 

Notably, our functional data did not reveal any significant differences regarding heparin versus citrate administration during RRT. Thus, our experiments rule out differences in leukocyte recruitment as a cause for the formerly observed increased susceptibility to infections in patients undergoing CVVH with regional citrate administration in comparison to systemic heparin treatment, as receptor expression profiles, slow leukocyte rolling and chemokine-dependent leukocyte adhesion were equally aberrant in both CVVH groups. It thus appears that the primary steps of the leukocyte-adhesion cascade are not affected by mode of anticoagulation during RRT; however, it is likely that—regarding the combatting of infections—other leukocyte effector functions might be affected. Indeed, recent work demonstrated that leukocyte degranulation is reduced in citrate-based RRT protocols [6]. Additionally, another study demonstrated that citrate affects neutrophil degranulation, and speculated that this step in RRT is primarily calcium-reliant [28]. A 1985 study showed that sodium citrate and another calcium chelator, EGTA (ethylene glycol bis (beta-aminoethylether)—N,N, N’,N’-tetraacetic acid), inhibited neutrophil oxygen consumption and lysosomal release [29]. Interestingly though, the same study showed an effect only in zymosan-stimulated cells, whereas no effect could be observed in fMLP-stimulated cells [29]. It is well-known that calcium is necessary for proper antibacterial leukocyte functions [30,31]. Nonetheless, calcium derangement in RRT is restricted mostly to the extracorporeal circuit itself, as calcium is controlled and reconstituted. Another possible cause for citrate as a regulator of infection rates in RRT might be hypophosphatemia, which has been shown to equally impact leukocyte degranulation [32,33]. In comparison, heparin was shown to dampen leukocyte elastase release, fMLP-mediated responses, rolling and adhesion in a biphasic dose–response manner [34], whereas another study reports a direct dose-dependent inhibition of elastase release and aggregation of leukocytes [35,36]. Nonetheless, a 1983 study reported on the synergistic effects of heparin and fMLP; when PMNs were preincubated with heparin, increased degranulation could be observed [37]. It is thus apparent that multiple factors must be considered when interpreting leukocyte dysfunction in critically ill AKI patients: 1. Underlying condition, 2. Severity and duration of AKI, 3. Choice and initiation of RRT protocol, 4. Mode of anticoagulation, 5. Dosage of anticoagulant and 6. Aspects of leukocyte functionality under examination. Further studies are needed to examine the precise interplay of these conditions and reveal the best setting to mediate overshooting (→ sepsis) and dysfunctional (→ risk of infection) leukocyte responses. 

A previous study also reported reduced CD11b expression following low concentrations of citrate [38], an effect we could not observe in our setting, as CD11b levels were elevated in the citrate group on day 1 of CVVH. Differences in sample preparations and study setting might account for these observations. 

A 2016 study by Singbartl et al. in mice and in 13 patients with septic shock observed that renal-replacement therapy is incapable of reversing AKI-induced neutrophil dysfunction in vitro [39]. Our study indeed demonstrates that usage of renal-replacement therapy, independent of mode of anticoagulation, could not reverse the observed leukocyte dysfunction in AKI in critically ill patients. Singbartl and colleagues attributed these observations to plasma resistin levels, which could not be cleared by renal-replacement therapy, but did not differentiate between different RRT-based anticoagulation protocols [39].

## 5. Conclusions

In conclusion, our data emphasize the drastic impact of AKI in critically ill patients on leukocyte functionality, abrogating slow leukocyte rolling and chemokine-mediated leukocyte adhesion by differentially controlling surface expression levels. These findings are not affected by CVVH-administration or mode of anticoagulation, and therefore hint towards other causes for recently uncovered differences in infection rates in patients receiving different CVVH-anticoagulation strategies, possibly affecting leukocyte effector functions.

## Figures and Tables

**Table 1:** Patient characteristics.

	Regional Citrate	Systemic Heparin	Control
Number of individual patients	16	20	4
Age (Mean ± SEM)	62.29 ± 4.43	65.90 ± 2.97	32.67 ± 4.98
Gender (% female)	50.00%	45.00%	25%
90-d mortality	37.50%	30.00%	0

## Data Availability

Not applicable.
